# Global warming impairs the olfactory floral signaling in strawberry

**DOI:** 10.1186/s12870-023-04564-6

**Published:** 2023-11-08

**Authors:** Guaraci Duran Cordeiro, Stefan Dötterl

**Affiliations:** Department of Environment & Biodiversity, Paris-Lodron University of Salzburg, Hellbrunnerstr. 34, Salzburg, 5020 Austria

**Keywords:** Chemical communication, Climate change, Floral scent, Pollination, Crop

## Abstract

**Background:**

Global warming is expected to impact the chemical communication between flowering plants and their pollinators. Surprisingly, it is unknown whether and how temperature-induced changes in scent emission affect pollinator behavior. Strawberry (*Fragaria x ananassa*) is a plant primarily pollinated by bees and hoverflies, with the former group being particularly attracted to the floral scent they emit.

**Results:**

Using chemical analytical, electrophysiological, and behavioral approaches we tested whether temperature-induced shifts in floral scent of strawberry affect chemical communication with its main bee pollinators (*Apis mellifera*, *Bombus terrestris*, *Osmia bicornis*). While strawberry flowers in the optimum scenario released 10.4 ng/flower/hour, mainly *p*-anisaldehyde (81%) and seven other scent compounds, in the warmer scenario, the flowers did not emit any detectable scent. In the behavioral experiments, the pollinators were attracted by the scents of the optimum scenario.

**Conclusions:**

We predict that the absence of detectable scent emissions from strawberry plants grown under heat stress will reduce the attractiveness of the flowers to the bee pollinators. Our study raises important ecological and agricultural questions, as decreased attractiveness of flowers to pollinators might potentially lead to insufficient bee pollination, with potential negative consequences for ecosystem functioning and crop yields, particularly in regions reliant on bees as primary pollinators. Given that our study centered on bee pollinators, it is needed to conduct further research to evaluate the impact on hoverflies.

**Supplementary Information:**

The online version contains supplementary material available at 10.1186/s12870-023-04564-6.

## Background

The global Earth surface temperature is projected to increase by up to 5.7 °C by the end of the 21st century, with heatwaves at this scale becoming increasingly frequent [1]. This global warming has serious consequences for organisms, ecosystem functioning and food production [1], while scientists worldwide are trying to understand the ecological effects of global warming on species interactions [2, 3]. In plants, increased temperatures affect flower traits involved in communication with pollinators [4, 5]. Among such traits are scents, which are released into the atmosphere through biochemical processes and play a key role in the attraction of pollinators [6–10]. The emission of scents by flowers is genetically determined, but influenced by environmental factors [11, 12]. Flowers can emit a large array of scent compounds from several chemical classes (e.g., terpenoids, aromatic compounds) [13]. Temperature affects the vaporization of floral scent compounds as well as the physiological processes involved in the biosynthesis and release of scents. Its effects differ among compounds and species, resulting in species-specific impacts of temperature on qualitative and quantitative scent properties [5, 14–17].

It is known that quantitative and qualitative changes in floral scent emissions have effects on the attractiveness of the volatile signal to pollinators [4, 18, 19]. Indeed, the amount of flower scent released must be enough to reach at least the threshold to be detected by pollinators, whereas too small amounts of scents cause inefficient pollinator attraction [20, 21]. It has been shown that flowers with higher floral scent emissions are often preferred by pollinators over flowers with lower emissions [22, 23]. Qualitative changes in scent bouquets increase the probability that pollinators do not find the flowers [18, 24]. Surprisingly, it is unknown whether and how temperature-induced changes in scent emission affect pollinator behavior and thus pollination in natural and agricultural settings.

Here, we used a combination of chemical analytical, electrophysiological, and behavioral approaches to quantify the effects of increased air temperatures on floral scent emissions of strawberry (*Fragaria x ananassa* Duch - Rosaceae), and to test for the olfactory attractiveness of floral scents to its main bee pollinators (*Apis mellifera* Linnaeus, *Bombus terrestris* Linnaeus, *Osmia bicornis* Linnaeus) [25]. Strawberry is an economically important crop cultivated worldwide [26], has hermaphroditic and female flowers, and pollination by bees and hoverflies increases yield and results in fruits with high quality [25, 27–29]. In this study, our primary focus was on bee pollinators that are particularly attracted by floral scent.

## Results and discussion

Our results showed that temperature strongly affected the floral scent. It was only detected from plants grown at optimum temperatures, whereas plants grown at warmer temperature did not release any detectable floral scent compounds. Plants grown at optimum temperatures emitted in total 10.45 ± 2.29 ng scent/flower/hour. We detected eight compounds (five aromatics compounds, two irregular terpenes, one sesquiterpene), of which aromatic *p*-anisaldehyde strongly dominated the samples (81,9%) (Table 1). All other compounds contributed < 5% to the scent discharge. The amount of floral scent emitted by strawberry grown at optimum temperatures was in between the amounts recorded from other strawberry crop varieties, such as Darselect, Elsanta, Honeoye, and Sonata (3 to 69 ng scent/flower/hour, [30, 31]) and from wild *Fragaria virginiana* (2 ng scent/flower/hour, [32]), one of the parental species of strawberry crops. However, the variety used in the present study obviously released a smaller number of compounds than wild *F. virginiana* (38 compounds, [32]). Other crop varieties also released smaller numbers of compounds (11–24; [30, 31]) than the wild species, but more than the variety studied here. Overall, the trend that modern crops emit fewer floral scents than wild relatives [33, 34] is also true for strawberry. Most compounds recorded in the present study are widespread among bee-pollinated flowers, with only benzyl tiglate and the ionones being less frequently recorded [13]. With the exception of *α*-ionone, all compounds identified in the present study were also released from other crop varieties or the wild strawberry, however, the variety studied here emitted higher relative amounts of *p*-anisaldehyde than other varieties/species. Dominant compounds in the floral scent of other crop varieties were benzaldehyde, nonanal and (*E*,*E*)-*α*-farnesene [30] or limonene [31], and of wild *F. virginiana* benzaldehyde, *α*-pinene, and benzyl alcohol [32].

**Table 1:** Total absolute amount of floral scent (mean ± standard error) and relative amount of the different floral scent compounds (%; mean ± standard error) emitted by strawberry plants (*N* = 10) grown under optimum temperature conditions. No scent was detected in samples collected from plants grown at increased temperatures. Compounds are listed according to chemical class. They were identified based on mass spectra and retention indices (KRI) of authentic standards, except *α*-ionone, which was identified based on mass spectrum and retention index of literature data

		Optimum
**Total absolute amount of scent (ng/flower/h)**		10.45 ± 2.29
**Compounds**	**KRI**	
***Aromatics***		
benzyl alcohol	1037	3.7 (± 1.1)
methyl salicylate	1205	4.3 (± 1.8)
*p*-anisaldehyde	1265	81.9 (± 5.8)
benzyl tiglate	1508	0.6 (± 0.3)
benzyl benzoate	1788	4.5 (± 1.8)
*Irregular terpenes*		
α-ionone	1436	2.3 (± 1.0)
dihydro-*β*-ionone	1442	0.6 (± 0.3)
*Sesquiterpenes*		
*(E,E)-α*-farnesene	1514	2.1 (± 1.4)

Our findings of a loss of detectable amounts of floral scent of plants grown at increased temperatures were unexpected, given that no previous study recorded changes in floral scent emissions to an extent found in the present study. Indeed, most studies only recorded changes in relative scent composition, with the presence of compounds being not influenced by temperature [35–37]. Only in one plant species (*Jasminum auriculatum*), a complete reduction in the emission of single floral scent compounds at high temperatures (35 °C) was recorded, while other compounds were still released [16]. The response of different crops to increased temperatures varies, for example, oilseed rape (*Brassica napus*) remained unaffected, whereas buckwheat (*Fagopyrum esculentum*) showed a threefold decrease in floral scent emission [17]. Thus, the floral scent emissions of strawberry crops are more sensitive to warmer temperature than the scent emissions of other plants.

**Fig. 1:**
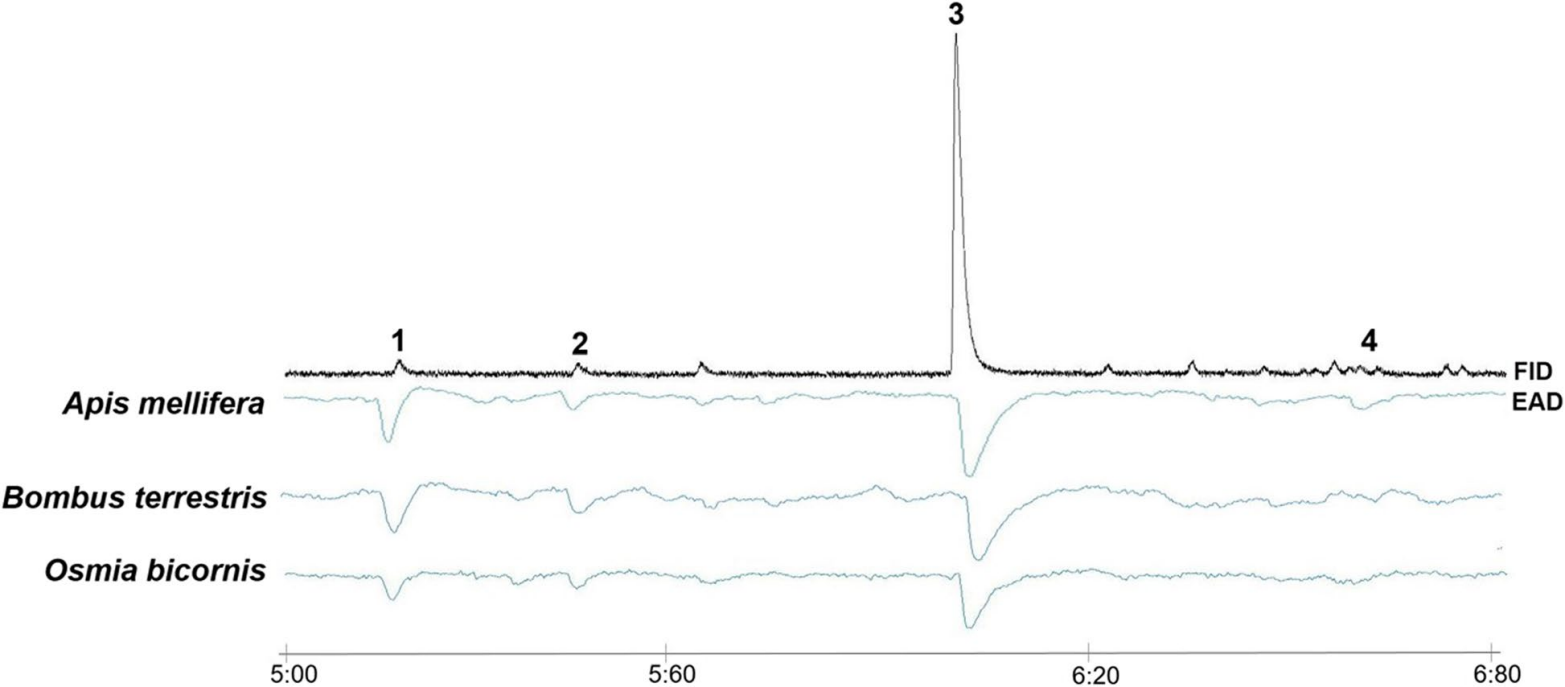
Examples of antennal responses (EADs) of *Apis mellifera*, *Bombus terrestris*, and *Osmia bicornis* to synthetic mixtures resembling the flower scent (FID) of strawberry (*Fragaria x ananassa*) under optimum scenario. (1) methyl salicylate, (2) benzyl alcohol, (3) *p*-anisaldehyde, (4) (*E*,*E*)-*α*-farnesene. Compounds not numbered were contaminants. The small differences in the times of EAD and FID responses are due to the chromatographic setup. This figure was provided by the authors, the FID and EADs were generated by the GC/EAD system and the authors added the species name

Our electrophysiological recordings revealed that all three pollinator species are sensitive to at least four of the eight flower scent compounds detected in the samples (Fig. 1; see also [38–40]). Benzyl tiglate and dihydro-*β*-ionone, which were not tested in our analyses, can also be sensed by bees, as previously shown by physiological measurements that tested the scent of apple flowers on honey bees [41] and synthetic strawberry flower scents on *O. bicornis* [30], respectively.

**Fig. 2:**
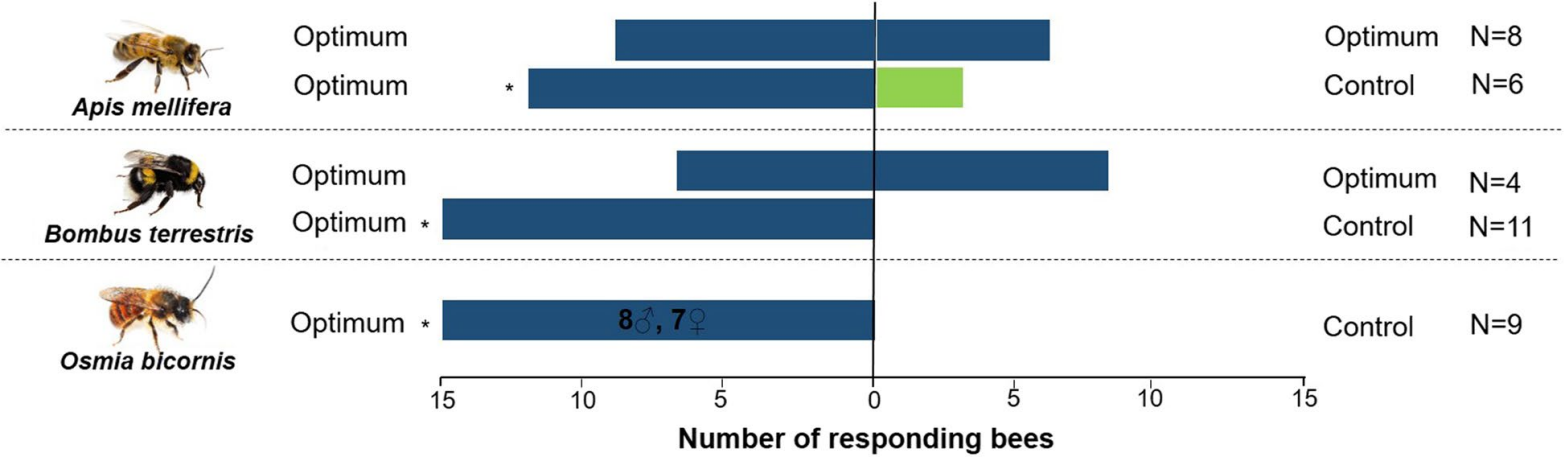
Number of bee individuals responding in dual-choice assays to synthetic mixtures resembling the flower scent of strawberry plants (*Fragaria x ananassa*) grown under optimum (blue) temperatures, and to negative controls (green) that consisted only of the solvent used to dilute the scent compounds. Workers of *Apis mellifera* and *Bombus terrestris*, and males and females of *Osmia bicornis* were used for the experiments. *: the two choices were differently attractive (p < 0.05) according to an exact binomial test of goodness-of-fit. *N* = number of replicates for each dual-choice assay (*O. bicornis*: 10 bees per replicate were placed in an indoor cage and assayed for 1 h; other species: free-flying bees in the outdoor flight-cage were observed for 1 h per replicate). This figure was provided by the authors

In the behavioral experiments, all three bees preferred scent from the optimum scenario against negative controls (Fig. 2). Two (*A. mellifera*, *B. terrestris*) of the three bee species were offered a same stimulus in a choice setting (scent of optimum scenario against scent of optimum scenario), and did not show a side bias. Therefore, the preference of the scent mixture when compared to negative controls were indeed due to the stimuli, and not due to a side bias of the bees (Fig. 2), showing that floral scents of strawberry crops are involved in the attraction of bee pollinators.

In a wild strawberry species, *F. virginiana*, which releases 1–2 ng of scent per hour and also produces hermaphrodite and female flowers, scent was also found to be important in pollinator attraction [29]. In this wild species, hermaphrodite flowers (ca. 1.8 ng/h) emitted slightly more scent than female flowers (ca. 1.4 ng/h), mainly due to the emission of 2-phenylethanol from stamens. This slight difference was enough that female flowers received in the field only half as many bee pollinator approaches as hermaphrodite flowers [32]. Thus, we predict that the absence of detectable scent emissions of cultivated strawberry grown under heat stress will reduce the attractiveness of the flowers to the bee pollinators. Even if bees might be attracted by visual flower cues alone [41–44], such cues typically attract flower visitors from only limited distances [45], and visual cues alone are often less attractive to bees then the combination of visual and olfactory cues [42–44]. Other pollinators, such as hoverflies, are sometimes primarily attracted by visual floral cues [46, 47] but see [48]. Thus, visually-guided pollinators could potentially sustain flower visits to strawberry flowers even in warmer conditions. Whether negative effects on scent emission might be counterbalanced by a temperature-induced higher activity of bees [49, 50], potentially resulting in the visitation of a higher number of flowers, is a topic for future research.

## Conclusion and implications

Our study shows for the first time that increased temperatures predicted by global warming (worst-case scenario) can result in a stop in the emission of detectable levels of floral scents. Future experiments might test whether scent emission gradually diminishes when temperature increases, at which temperature the plants stop releasing detectable amounts of floral scents, and whether different strawberry cultivars respond similar to increased temperatures. Our finding generally raises the question of whether modern crops, such as cultivated strawberry, will continue to be sufficiently attractive to pollinators. This question could be addressed in future research as well, and considering both bee and hoverfly pollinators of strawberry. Future research might also address the question to which extent increased temperatures affect pollinator attraction in natural settings and influence ecosystem functioning, and how increased temperatures will affect the activity of pollinators and their flower-visiting behavior. Currently, the molecular and biochemical processes in the biosynthesis and emission of floral scent affected by heat stress are mostly unknown [5, 16, 51], and therefore, more efforts are needed to elucidate the effects of increased temperatures on both floral scent production and floral scent emission at a molecular level. This will help to develop breeding programs with the aim to increase floral scent emissions at increased temperatures and to generate more heat-tolerant crops.

## Methods

### Crop species and pollinators

Strawberry (*Fragaria x ananassa* Duch – Rosaceae) is a fruit crop widely appreciated for its characteristic aroma and sweetness. Adequate bee pollination increases yield and fruit quality [30]. We used the main bee pollinators of strawberry for our experiments: *Apis mellifera* Linnaeus, 1758 (obtained from a local beekeeper), *Bombus terrestris* Linnaeus, 1758 (Biobest®, Belgium), and *Osmia bicornis* Linnaeus, 1758 (Mauerbienenzucht®, Germany) [25].

Strawberry is a gender-dimorphic species with hermaphroditic and female flowers, but in contrast to wild strawberry [32], we did not find an effect of flower sex on scent emission (total amount of scent: Z= -0.32, *N* = 10, *p* = 0.761; relative scent composition: Pseudo-F_1,9_=1.12, *p* = 0.323) and thus did not discriminate between the flower sexes in the present study.

### Crop plant cultivation and temperature regime

The strawberry plants were cultivated from seeds (variety - Florian F1, company - Dürr-Samen®) in two plant growth chambers (Liebherr, Profi line, Germany; adapted with a multistage temperature controller, model TAR 1700-2, Elreha, Germany, and light timer switch, model D21ASTRO 230 V 50/60 Hz, Legrand®, Germany) that differed in their temperature setting (optimum and increased, see below). The seeds were randomly assigned to one of the chambers and sown in pots (9 × 8 × 9 cm) using standard soil (Einheitserde®, Profi Substrat) and fertilized once with 50 ml/pot (Wufax®, Nitrogen: 12%, Phosphate: 4%, Potassium: 6%) when plants reached mid-age.

The requirements of strawberry plants for light conditions and water availability were controlled following literature data [52]. Plants were cultivated in both chambers at 16 h light/08 h darkness, and the light intensity was 2000 lx (via cool white led lamps, model VT-5959 LED-Flutlicht, V-TAC, 50 W). The air humidity ranged between 60% and 70%. To keep the soil at comparable moisture levels during the development of the plants and between the different treatments, as measured by a tensiometer (model FDA 602 TM2, ALMEMO®, Germany), the water supply varied according to the age of the plants and the temperature scenario. When they were sown, the amount was, independent of the scenario, 15 ml/plant/day, in the mid-ages 60 ml/plant/day (optimum scenario) and 90 ml/plant/day (warmer scenario), and, during the flowering phase, 120 ml/plant/day (optimum scenario) and 170 ml/plant/day (warmer scenario).

The plants were grown under two temperature scenarios: optimum and 5 °C higher than optimal temperatures (according to the global warming scenario SSP-8.5, [1]. The mean optimal temperature for the growth and flowering of cultivated strawberry (*F. ananassa*) plants is 20 °C [52]. Considering the mean daily thermal amplitude in Central Europe [53, 54], strawberry plants were cultivated in the optimal scenario at day and night with temperatures of 23 °C and 13 °C (mean 20 °C, when considering the length of the day and night periods), respectively, and in the warmer scenario, the temperatures were 28 °C and 18 °C (mean 25 °C) during day and night, respectively. For each temperature scenario, 12 individual plants were cultivated. Treatment did not have an effect on the number of flowers produced per individual (unpublished data). In both scenarios the plants and flowers developed normally.

### Sampling and analysis of flower scents

Sampling of scent samples was performed inside the growth chambers by dynamic headspace. From 12 individuals cultivated for each scenario, 10 individuals of the optimum scenario and nine individuals of the warmer scenario produced flowers during the experiment and were sampled. The samples were obtained from flowers at the beginning of their first day of anthesis, throughout the day depending on flower opening time, which ranged from 8:20 AM to 4:15 PM. A single flower per sample was enclosed in a polyester oven bag (Toppits®). After bagging, two small adsorbend tubes were inserted into the bag: one was used to trap the floral scent, and the other (glass vial filled with 5 mg Carbotrap B) was used to insert clean air from outside of the growth chambers to avoid internal air contamination). The samplings lasted 30 min using membrane pumps (G12/01 EB; Gardner Denver Thomas GmbH, Fürstenfeldbruck, Germany). This time period was enough to obtain the maximum number of compounds as determined by preliminary analyses that used sampling times between 15 min and 2 h. The flows of both pumps were adjusted at 200 ml/min with the help of flowmeters. The adsorbend tubes (quartz vials, length: 25 mm, inner diameter: 2 mm) were filled with 1.5 mg Tenax-TA (mesh 60–80) and 1.5 mg Carbotrap B (mesh 20–40, both Supelco). The adsorbends were fixed in the tubes using glass wool. Dynamic headspace samples of green leaves (*N* = 3 samples per scenario) were collected with the same method to discriminate between vegetative (not considered for subsequent analyses) and flower-specific scent components. Samples from empty oven bags (*N* = 3) inside the growth chambers were collected to identify potential contaminants.

Scent samples were analyzed using GC/MS (gas chromatography/mass spectrometry). The system consisted of an automated thermal desorption system (model TD-20, Shimadzu, Japan) coupled to a QP2010 Ultra EI GC/MS (Shimadzu, Japan) equipped with a Zebron™ ZB-5 fused silica column (5% phenyl 95% dimethylpolysiloxane; 60 m long; inner diameter 0.25 mm; film thickness 0.25 μm; Phenomenex), as described previously [55]. The GC/MS data were processed using GCMSsolution (Version 4.41, Shimadzu Corporation 2015). The tentative identification of compounds was carried out by the authors using the mass spectral libraries Wiley 9, Nist 2011/FFNSC 2, and [56], as well as the database available in MassFinder 3. The identity of all compounds was confirmed by a comparison of mass spectra and retention times with those of authentic standard compounds available at the Plant Ecology lab of the Paris-Lodron University of Salzburg. To determine the amount of scent trapped, known amounts of monoterpenes, aliphatics, and aromatics were added to clean adsorbent tubes and analyzed by GC/MS as described above; mean peak areas (total ion current) of these compounds were used to determine the total amount of strawberry scent [57].

### Synthetic scent sample

A synthetic mixture with four compounds (benzyl alcohol, methyl salicylate, *p*-anisaldehyde, (*E*,*E*)-*α*-farnesene), which explained in the mean > 92% of the total floral scents collected from plants grown at the optimum scenario (no compounds were detected from plants grown at increased temperatures; see Results), was prepared to be used for electroantennographic analyses. Given that the three bee species responded in the electrophysiological measurements to all the compounds included in this mixture (see Results), the same mixture was also used for behavioral experiments (see below). When pipetted on filter papers (diameter 1 cm; Whatman) used for behavioral experiments, sampled by dynamic headspace, and analyzed by GC/MS, this mixture resembled the relative and absolute composition of these compounds in the natural samples (Supplementary Information Fig. S1). The synthetic scent mixture was prepared with compounds available in the reference collection of the Plant Ecology lab of the Paris-Lodron University of Salzburg in the highest purity available (> 90%). The solvent to dilute the compounds and used as a negative control in behavioral experiments (see below) was acetone (Sigma-Aldrich, 99.8%).

The other compound detected in the floral scent samples were not included in the synthetic mixture as they were found only in less than half of the flower scent samples (benzyl tiglate) or were identified just after the physiological and behavioral experiments have been performed (ionones, benzyl benzoate).

### Electroantennographic detection

The synthetic scent mixture was tested on the antennae of the three bee pollinators (*N* = 7 worker bee individuals each of *A. mellifera* and *B. terrestris*, and 5 females and 4 males of *O. bicornis*) by GC/EAD (gas chromatography coupled with electroantennographic detection) to evaluate the compounds eliciting antennal responses. The GC/EAD system, the same as that used by [58], consisted of a gas chromatograph (Agilent 7890 A, Santa Clara, California, USA) equipped with a flame ionization detector (FID) and an EAD setup (heated transfer line, 2-channel USB acquisition controller) provided by Syntech (Kirchzarten, Germany). A volume of 1 µl of the samples was injected (temperature of injector: 250 °C) splitless at 40 °C oven temperature, followed by opening the split vent after 0.5 min and heating the oven at a rate of 10 °C min^−1^ to 220 °C. A DMT Beta SE column (30 m long, inner diameter 0.25 mm, film thickness 0.23 μm, MEGA-DEX) was used for the analyses, and the column flow (carrier gas: hydrogen) was set at 3 ml min^−1^. The column was split at the end by a µFlow splitter (Gerstel, Mülheim, Germany) into two deactivated capillaries leading to the FID (2 m x 0.15 μm) and EAD (1 m x 0.2 μm) setups. Makeup gas (N_2_) was introduced in the splitter at 25 ml min^−1^. The outlet of the EAD was placed in a cleaned and humidified airflow that was directed over the antenna of the pollinators. The antennae were cut at their base and tip, inserted between two electrodes filled with an insect ringer (8.0 g/l NaCl, 0.4 g/l KCl, 0.4 g/l CaCl_2_), and connected to silver wires as described previously [59].

A floral compound was considered EAD-active in a bee species when it elicited a depolarization response in at least four individuals.

### Behavioral experiments

Behavioral assays were performed to test whether the synthetic flower scent of strawberry plants is capable of attracting strawberry-naïve bee pollinators. These assays were conducted outdoors (*A. mellifera*, *B. terrestris*) and indoors (*O. bicornis*) at the Paris-Lodron University of Salzburg. Tests with *O. bicornis* were performed indoors between May and June 2021, as weather conditions during their flight period did not allow testing outdoors.

The outdoor behavioral experiments were performed between June and August 2021 in a flight cage (wooden construction clamped with white gauze of 8 × 4 × 2.2 m) in the Botanical Garden, the same as that successfully used with bees before [41]. In this flight cage, we kept the bees (*A. mellifera* hive, obtained from a local beekeeper, with 10 combs; *B. terrestris* hive, obtained from Biobest®, Belgium), and there was *Reseda lutea* (Resedaceae) continuously flowering and used as a pollen and nectar resource. Scent samples were offered on artificial flowers in dual-choice assays, with a distance of 1.50 m between them. The artificial flowers were made of blue bond paper of 7 cm diameter and tied on wooden sticks. White filter paper of 1 cm diameter (Whatman), onto which a scent sample was applied, was placed in the middle of the artificial flower (Supplementary Information Fig. S2A and S2B).

The indoor behavioral experiments with *O. bicornis* (obtained from Mauerbienenzucht®, Germany) were performed in a small cage (30 × 30 × 30 cm) placed in an experimental room (temperature: 25 °C) that was illuminated by five T26 EVG Grolux lamps. For each experimental run (17 runs in total), we tested 10 individuals per time (males and females separately) for one hour. Bees were fed sugar water (50%, w/w) inside the cage in a small pot. The samples were offered in dual-choice assays on filter papers (diameter 3.7 cm; Whatman) placed on aluminum foil on the bottom of the cage with a distance of 20 cm between them (Supplementary Information Fig. S2C and S2D).

For each pollinator, we tested the synthetic scent mixture against a negative control (acetone). To test for a potential side bias, the synthetic scent mixture was also tested against itself (only for outdoor tests).

For all assays, 150 µl of the synthetic mixture /acetone was used. As determined by GC/MS of headspace samples collected at ambient temperature, the absolute amount of scent offered to pollinators was equivalent to the scent of 100 flowering plant individuals, i.e., representing a small crop area (Supplementary Information Table S1). Thus, the scent offered to the bees was in a natural range, as a bee that approaches a strawberry crop field might easily be exposed to the headspace of 100 individuals. One bioassay trial lasted for 1 h, whereas the position of the artificial flowers/filter papers were changed and the scent mixture was renewed after 30 min. Bees that landed on artificial flower/filter paper were recorded and marked with a nontoxic pen (Posca® - Tokyo, Japan) to avoid counting an individual bee twice. A specific assay was replicated (one trial = one replicate) until a minimum of 15 bees responded. Overall, we performed bioassays on a total of 16 days, for in total 38 h (outdoor: *A. mellifera* − 6 days/14 hours, *B. terrestris* − 5 days/15 hours; indoor: *O. bicornis* − 5 days/9 hours).

### Data analysis

The dual-choice behavioral experiments (number of bees responding to the different choices) were analyzed by exact binomial tests of goodness-of-fit using the spreadsheet provided by http://udel.edu/~mcdonald/statexactbin.html. We tested the null hypothesis that the synthetic scent mixture was equally attractive as the negative control, and that the two sides attracted the same number of bees when offering the same stimulus at the two sides.

### Supplementary Information

**Additional file 1: Table S1.** The amount (mg) of compounds contained in 150 µl of synthetic scent mixtures that were used for behavioral assays. The headspace of these mixtures resembled the floral scent emissions of 100 strawberry individuals (c. 400 flowers, as plants had in the mean four flowers that were open simultaneously) for optimum scenarios.** Fig S1.** Comparisons of chromatograms of flower scent emission in strawberry (*Fragaria x ananassa*) under optimum scenario with their respective synthetic scent mixture (green line). 1. benzyl alcohol; 2. methyl salicylate; 3. *p*-anisaldehyde; 4. (*E*,*E*)-*α*-farnesene. Scent compounds not numbered were vegetative components or contaminants. **Fig S2.** A-The flight cage in the Botanical Garden of the Paris-Lodron University of Salzburg. B-Set up of the behavioral experiments in the flight cage using artificial flowers and a *Bombus terrestris* bee on an artificial flower (inset photo); C- the small cage used indoors for behavioral assays; D- setup of indoor behavioral assays. For more details, see Methods.

## Data Availability

The authors declare that the data supporting the findings of this study are available within the paper and its supplementary information files.

## References

[1] IPCC. Climate Change 2022: Impacts, Adaptation and Vulnerability. Contribution of Working Group II to the Sixth Assessment Report of the Intergovernmental Panel on Climate Change. In: Pörtner H-O, Roberts DC, Tignor M, Poloczanska ES, Mintenbeck K, Alegría A, Craig M, Langsdorf S, Löschke S, Möller V, Okem A, Rama B, editors. Cambridge and New York: Cambridge University Press; 2022. p. 3056.

[2] Thackeray SJ, Henrys PA, Hemming D, Bell JR, Botham MS, Burthe S (2016). Phenological sensitivity to climate across taxa and trophic levels. Nature.

[3] Zamora-Gutierrez V, Rivera-Villanueva AN, Martinez Balvanera S, Castro‐Castro A, Aguirre‐Gutiérrez J (2021). Vulnerability of bat-plant pollination interactions due to environmental change. Glob Change Biol.

[4] Burkle LA, Runyon JB (2017). The smell of environmental change: using floral scent to explain shifts in pollinator attraction. Appl Plant Sci.

[5] Borghi M, Perez de Souza L, Yoshida T, Fernie AR (2019). Flowers and climate change: a metabolic perspective. New Phytol.

[6] Jakobsen HB, Olsen CE (1994). Influence of climatic factors on emission of flower volatiles in situ. Planta.

[7] Dobson HEM, Dudareva N, Pichersky E (2006). Relationship between floral fragrance composition and type of pollinator. Biology of floral scent.

[8] Wiley RH (2020). Signal detection and animal communication. Adv Study Behav.

[9] Raguso RA (2008). Wake up and smell the roses: the ecology and evolution of floral scent. Annu Rev Ecol Evol Syst.

[10] Cordeiro GD, Pinheiro M, Dötterl S, Alves-dos-Santos I (2017). Pollination of *Campomanesia phaea* (Myrtaceae) by night-active bees: a new nocturnal pollination system mediated by floral scent. Plant Biol.

[11] Van Schie CCN, Haring MA, Schuurink RC (2006). Regulation of terpenoid and benzenoid production in flowers. Curr Opin Plant Biol.

[12] Pichersky E, Gang DR (2000). Genetics and biochemistry of secondary metabolites in plants: an evolutionary perspective. Trends Plant Sci.

[13] Knudsen JT, Eriksson R, Gershenzon J, Ståhl B (2006). Diversity and distribution of floral scent. Bot Rev.

[14] Sagae M, Oyama-Okubo N, Ando T, Marchesi E, Nakayama M (2008). Effect of temperature on the floral scent emission and endogenous volatile profile of *Petunia axillaris*. Biosci Biotechnol Biochem.

[15] Farré-Armengol G, Fernández-Martínez M, Filella I, Junker RR, Peñuelas J (2020). Deciphering the biotic and climatic factors that influence floral scents: a systematic review of floral volatile emissions. Front Plant Sci.

[16] Barman M, Mitra A. Floral maturation and changing air temperatures influence scent volatiles biosynthesis and emission in *Jasminum Auriculatum *Vahl. Environ Exp Bot. 2021;181:104296.

[17] Cordeiro GD, Dötterl S (2023). Floral scents in bee-pollinated buckwheat and oilseed rape under a global warming scenario. Insects.

[18] Beyaert I, Hilker M (2014). Plant odour plumes as mediators of plant-insect interactions. Biol Rev.

[19] Larue AAC, Raguso RA, Junker RR (2016). Experimental manipulation of floral scent bouquets restructures flower–visitor interactions in the field. J Anim Ecol.

[20] Majetic CJ, Raguso RA, Ashman TL (2009). The sweet smell of success: floral scent affects pollinator attraction and seed fitness in *Hesperis matronalis*. Funct Ecol.

[21] Arpaia S, De Cristofaro A, Guerrieri E, Bossi S, Cellini F, Di Leo GM, Germinara GS, Iodice L, Maffei ME, Petrozza A, Sasso R, Vitagliano S (2011). Foraging activity of bumblebees (*Bombus terrestris* L.) on Bt-expressing eggplants. Arthropod Plant Interact.

[22] Kessler D, Gase K, Baldwin IT (2008). Field experiments with transformed plants reveal the sense of floral scents. Science.

[23] Parachnowitsch AL, Raguso RA, Kessler A (2012). Phenotypic selection to increase floral scent emission, but not flower size or colour in bee-pollinated *Penstemon digitalis*. New Phytol.

[24] Morse A, Kevan P, Shipp L, Khosla S, McGarvey B (2012). The impact of greenhouse tomato (Solanales: Solanaceae) floral volatiles on bumble bee (Hymenoptera: Apidae) pollination. Environ Entomol.

[25] Klein A, Vaissiere BE, Cane JH, Steffan-Dewenter I, Cunningham SA, Kremen C, Tscharntke T (2007). Importance of pollinators in changing landscapes for world crops. Proc R Soc B Biol Sci.

[26] FAOSTAT. http://faostat3.fao.org/. 2014. Accessed 30 Jan 2022.

[27] Klatt BK, Holzschuh A, Westphal C, Clough Y, Smit I, Pawelzik E, Tscharntke T (2014). Bee pollination improves crop quality, shelf life and commercial value. Proc R Soc B Biol Sci.

[28] Abrol DP, Gorka AK, Ansari MJ, Al-Ghamdi A, Al-Kahtani S (2019). Impact of insect pollinators on yield and fruit quality of strawberry. Saudi J Biol Sci.

[29] Najberek K, Kosior A, Solarz W (2021). Alien balsams, strawberries and their pollinators in a warmer world. BMC Plant Biol.

[30] Klatt BK, Burmeister C, Westphal C, Tscharntke T, von Fragstein M (2013). Flower volatiles, crop varieties and bee responses. PLoS ONE.

[31] Ceuppens B, Ameye M, Van Langenhove H, Roldan-Ruiz I, Smagghe G (2015). Characterization of volatiles in strawberry varieties ‘Elsanta’ and ‘Sonata’ and their effect on bumblebee flower visiting. Arthropod Plant Interac.

[32] Ashman TL, Bradburn M, Cole DH, Blaney BH, Raguso RA (2005). The scent of a male: the role of floral volatiles in pollination of a gender dimorphic plant. Ecology.

[33] Young AM, Severson DW (1994). Comparative analysis of steam distilled floral oils of cacao cultivars (*Theobroma cacao* L., Sterculiaceae) and attraction of flying insects - implications for a *Theobroma* pollination syndrome. J Chem Ecol.

[34] Ferrari MJ, Stephenson AG, Mescher MC, De Moraes CM (2006). Inbreeding effects on blossom volatiles in *Cucurbita pepo* subsp. texana (Cucurbitaceae). Am J Bot.

[35] Farré-Armengol G, Filella I, Llusià J, Niinemets Ü, Peñuelas J (2014). Changes in floral bouquets from compound-specific responses to increasing temperatures. Glob Change Biol.

[36] Hu Z, Zhang H, Leng P, Zhao J, Wang W, Wang S (2013). The emission of floral scent from *Lilium* ‘siberia’ in response to light intensity and temperature. Acta Physiol Plant.

[37] Cna’ani A, Muehlemann JK, Ravid J, Masci T, Klempien A, Nguyen TT, Dudareva N, Pichersky E, Vainstein A (2015). *Petunia × hybrida* floral scent production is negatively affected by high-temperature growth conditions. Plant Cell Environ.

[38] Wadhams LJ, Blight MM, Kerguelen V, Le Métayer M, Marion-Poll F, Masson C (1994). Discrimination of oilseed rape volatiles by honey bee: novel combined gas chromatographic-electrophysiological behavioral assay. J Chem Ecol.

[39] Dötterl S, Vereecken N (2010). The chemical ecology and evolution of bee-flower interactions: a review and perspectives. Can J Zool.

[40] Burger H, Ayasse M, Dötterl S, Kreissl S, Galizia CG (2013). Perception of floral volatiles involved in host-plant finding behaviour: comparison of a bee specialist and generalist. J Comp Physiol.

[41] Rachersberger M, Cordeiro GD, Schäffler I, Dötterl S (2019). Honeybee pollinators use visual and floral scent cues to find apple (*Malus domestica*) flowers. J Agri Food Chem.

[42] Burger H, Dötterl S, Ayasse M (2010). Host-plant finding and recognition by visual and olfactory floral cues in an oligolectic bee. Funct Ecol.

[43] Milet-Pinheiro P, Ayasse M, Schlindwein C, Dobson HE, Dötterl S (2012). Host location by visual and olfactory floral cues in an oligolectic bee: innate and learned behavior. Behav Ecol.

[44] Dötterl S, Milchreit K, Schäffler I (2011). Behavioural plasticity and sex differences in host finding of a specialized bee species. J Comp Physiol.

[45] Streinzer M, Paulus HF, Spaethe J (2009). Floral colour signal increases short-range detectability of a sexually deceptive orchid to its bee pollinator. J Exp Biol.

[46] Sutherland JP, Sullivan MS, Poppy GM (1999). The influence of floral character on the foraging behaviour of the hoverfly, *Episyrphus Balteatus*. Entomol Exp Appl.

[47] Laubertie EA, Wratten SD, Sedcole JR (2006). The role of odour and visual cues in the pan-trap catching of hoverflies (Diptera: Syrphidae). Ann Appl Biol.

[48] Primante C, Dötterl S (2010). A syrphid fly uses olfactory cues to find a non-yellow flower. J Chem Ecol.

[49] Blois JL, Zarnetske PL, Fitzpatrick MC, Finnegan S (2013). Climate change and the past, present, and future of biotic interactions. Science.

[50] Giannini TC, Costa WF, Cordeiro GD, Imperatriz-Fonseca VL, Saraiva AM, Biesmeijer J, Garibaldi LA (2017). Projected climate change threatens pollinators and crop production in Brazil. PLoS ONE.

[51] Dudareva N, Klempien A, Muhlemann JK, Kaplan I (2013). Biosynthesis, function and metabolic engineering of plant volatile organic compounds. New Phytol.

[52] Hytönen T, Kurokura T (2020). Control of flowering and runnering in strawberry. Hort J.

[53] PlavcovÁ E, KyselÝ J (2011). Evaluation of daily temperatures in Central Europe and their links to large-scale circulation in an ensemble of regional climate models. Tellus A Dyn Meteorol Oceanogr.

[54] Worldclim. New 1-km spatial resolution climate surfaces for global land areas. Available at http://worldclim.org/version2 . 2020. Accessed 30 Jan 2022.

[55] Mitchell TC, Dötterl S, Schaefer H (2015). Hawkmoth pollination and elaborate petals in Cucurbitaceae: the case of the Caribbean endemic *Linnaeosicyos amara*. Flora.

[56] Adams RP (2007). Identification of essential oil components by gas chromatography/mass spectrometry.

[57] Cordeiro GD, Santos IGF, Silva CI, Schlindwein C, Alves-dos-Santos I, Dötterl S (2019). Nocturnal floral scent profiles of Myrtaceae fruit crops. Phytochemistry.

[58] Heiduk A, Brake I, von Tschirnhaus M, Göhl M, Jürgens A, Johnson SD, Meve U, Dötterl S (2016). *Ceropegia sandersonii* mimics attacked honey bees to attract kleptoparasitic flies for pollination. Curr Biol.

[59] Dötterl S, Füssel U, Jürgens A, Aas G (2005). 1,4-Dimethoxybenzene, a floral scent compound in willows that attracts an oligolectic bee. J Chem Ecol.

