# Supplementary material for: Global warming impairs the olfactory floral signaling in strawberry
Source: BMC Plant Biol. 2023 Nov 8;23:549. doi: 10.1186/s12870-023-04564-6 (PMC10631152; doi:10.1186/s12870-023-04564-6)
Supplement: Supplementary file 1 — Additional file 1: Table S1. The amount (mg) of compounds contained in 150 µl of synthetic scent mixtures that were used for behavioral assays. The headspace of these mixtures resembled the floral scent emissions of 100 strawberry individuals (c. 400 flowers, as plants had in the mean four flowers that were open simultaneously) for optimum scenarios. Fig S1. Comparisons of chromatograms of flower scent emission in strawberry (Fragaria x ananassa) under optimum scenario with their respective synthetic scent mixture (green line). 1. benzyl alcohol; 2. methyl salicylate; 3. p-anisaldehyde; 4. (E,E)-α-farnesene. Scent compounds not numbered were vegetative components or contaminants. Fig S2. A-The flight cage in the Botanical Garden of the Paris-Lodron University of Salzburg. B-Set up of the behavioral experiments in the flight cage using artificial flowers and a Bombus terrestris bee on an artificial flower (inset photo); C- the small cage used indoors for behavioral assays; D- setup of indoor behavioral assays. For more details, see Methods. [file 12870_2023_4564_MOESM1_ESM.docx]

**Supplementary Information**

**BMC Plant Biology**

**Global warming impairs the olfactory floral signaling in strawberry**

Guaraci D. Cordeiro^1^*, Stefan Dötterl^1^

^1^Department of Environment & Biodiversity, Paris‐Lodron University of Salzburg, Hellbrunnerstr. 34, 5020, Salzburg, Austria. *Corresponding author: guaradc@gmail.com

**Table S1.** The amount (mg) of compounds contained in 150 µl of synthetic scent mixtures that were used for behavioral assays. The headspace of these mixtures resembled the floral scent emissions of 100 strawberry individuals (c. 400 flowers, as plants had in the mean four flowers that were open simultaneously) for optimum scenarios.

|  | **Optimum-scent** |
| --- | --- |
| *p*-anisaldehyde | 0.01612 |
| benzyl alcohol | 0.00015 |
| methyl salicylate | 0.00017 |
| (*E*,*E*)-*α*-farnesene | 0.00024 |
| acetone | 117.58823 |


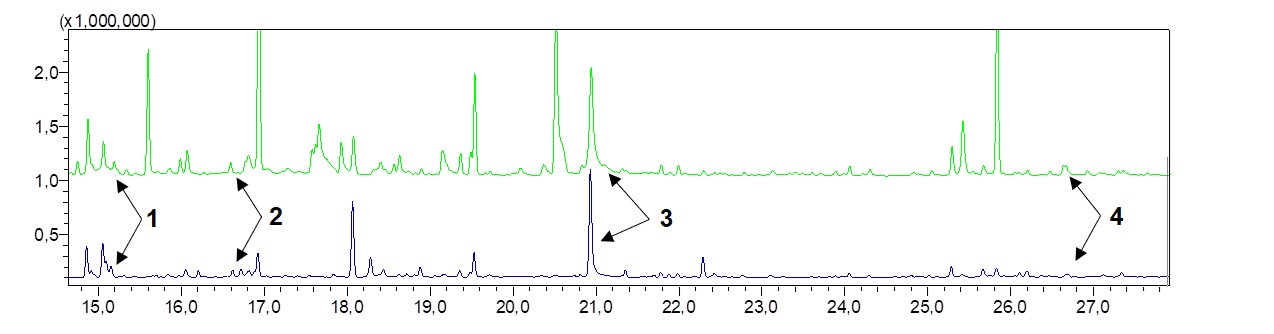


**Fig S1.** Comparisons of chromatograms of flower scent emission in strawberry (*Fragaria x ananassa*) under optimum scenario with their respective synthetic scent mixture (green line). 1. benzyl alcohol; 2. methyl salicylate; 3. *p*-anisaldehyde; 4. (*E*,*E*)-*α*-farnesene. Scent compounds not numbered were vegetative components or contaminants.


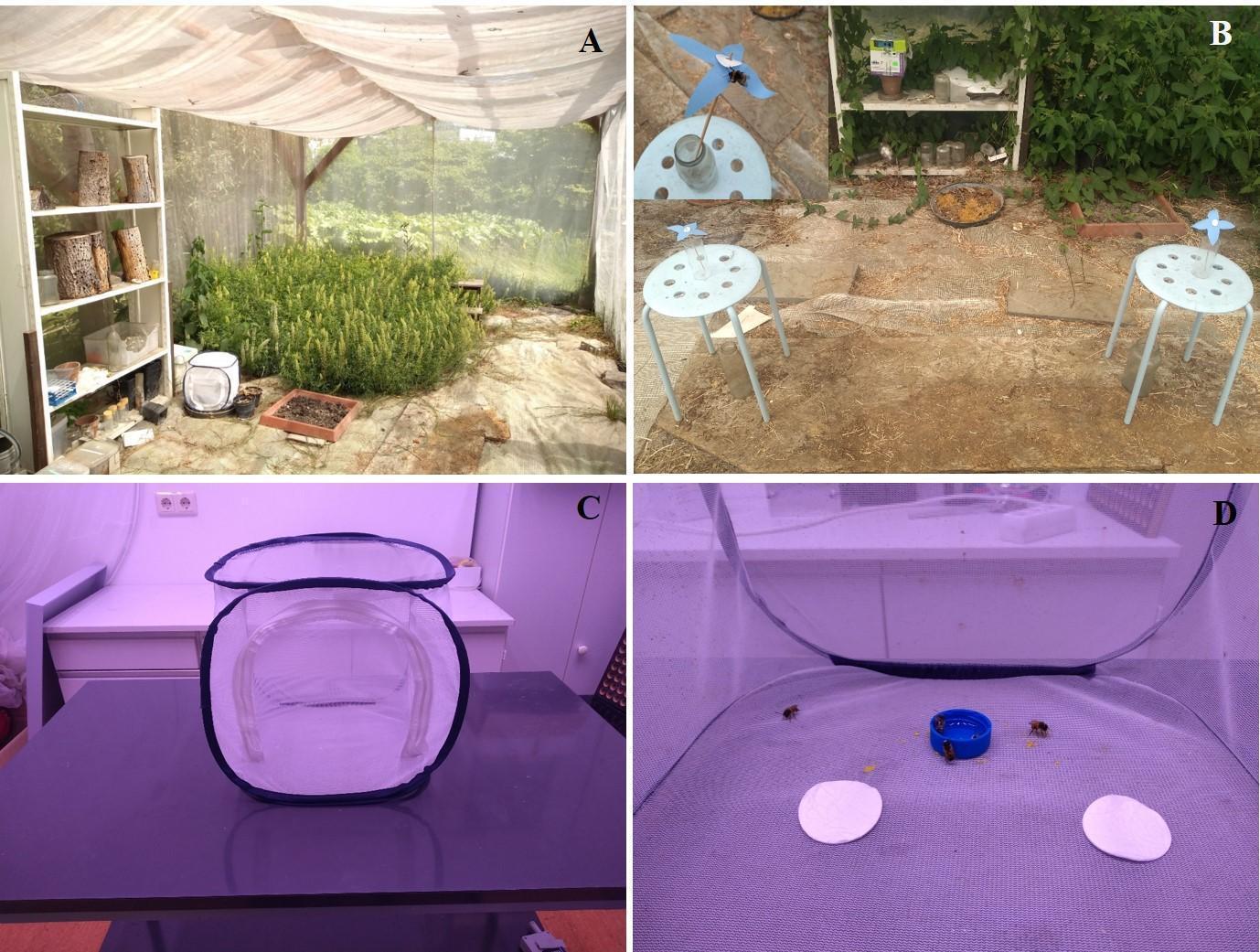


**Fig S2.** A-The flight cage in the Botanical Garden of the Paris-Lodron University of Salzburg. B-Set up of the behavioral experiments in the flight cage using artificial flowers and a *Bombus terrestris* bee on an artificial flower (inset photo); C- the small cage used indoors for behavioral assays; D- setup of indoor behavioral assays. For more details, see Methods.
